# Supplementary figures and images for: A Previously Undescribed Highly Prevalent Phage Identified in a Danish Enteric Virome Catalog
Source: mSystems. 2021 Oct 19;6(5):e00382-21. doi: 10.1128/mSystems.00382-21 (PMC8525569; doi:10.1128/mSystems.00382-21)

A

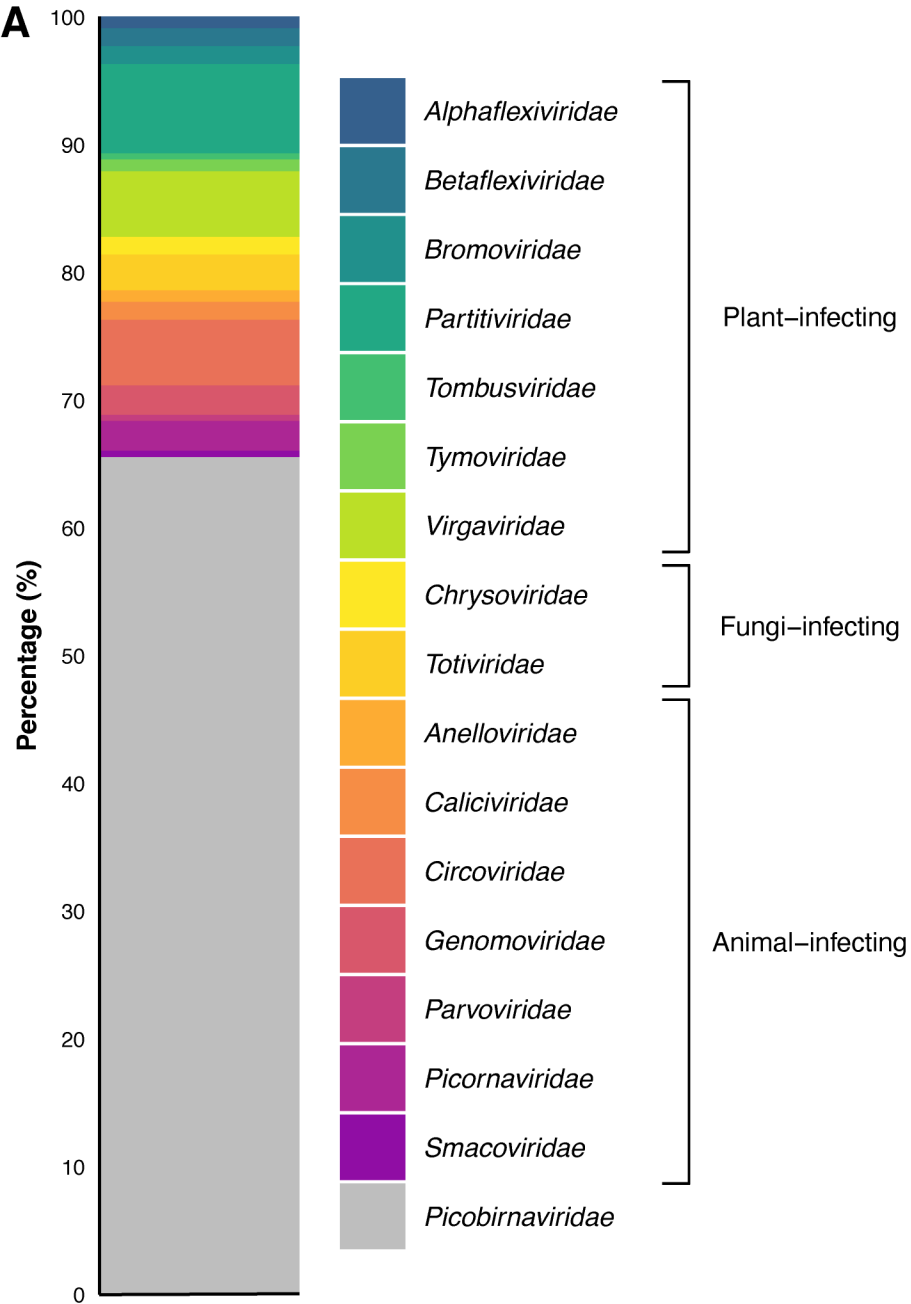

B

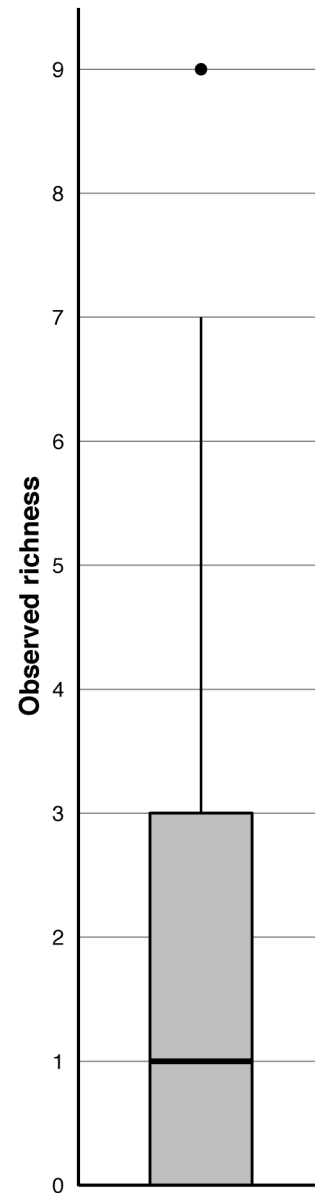

C

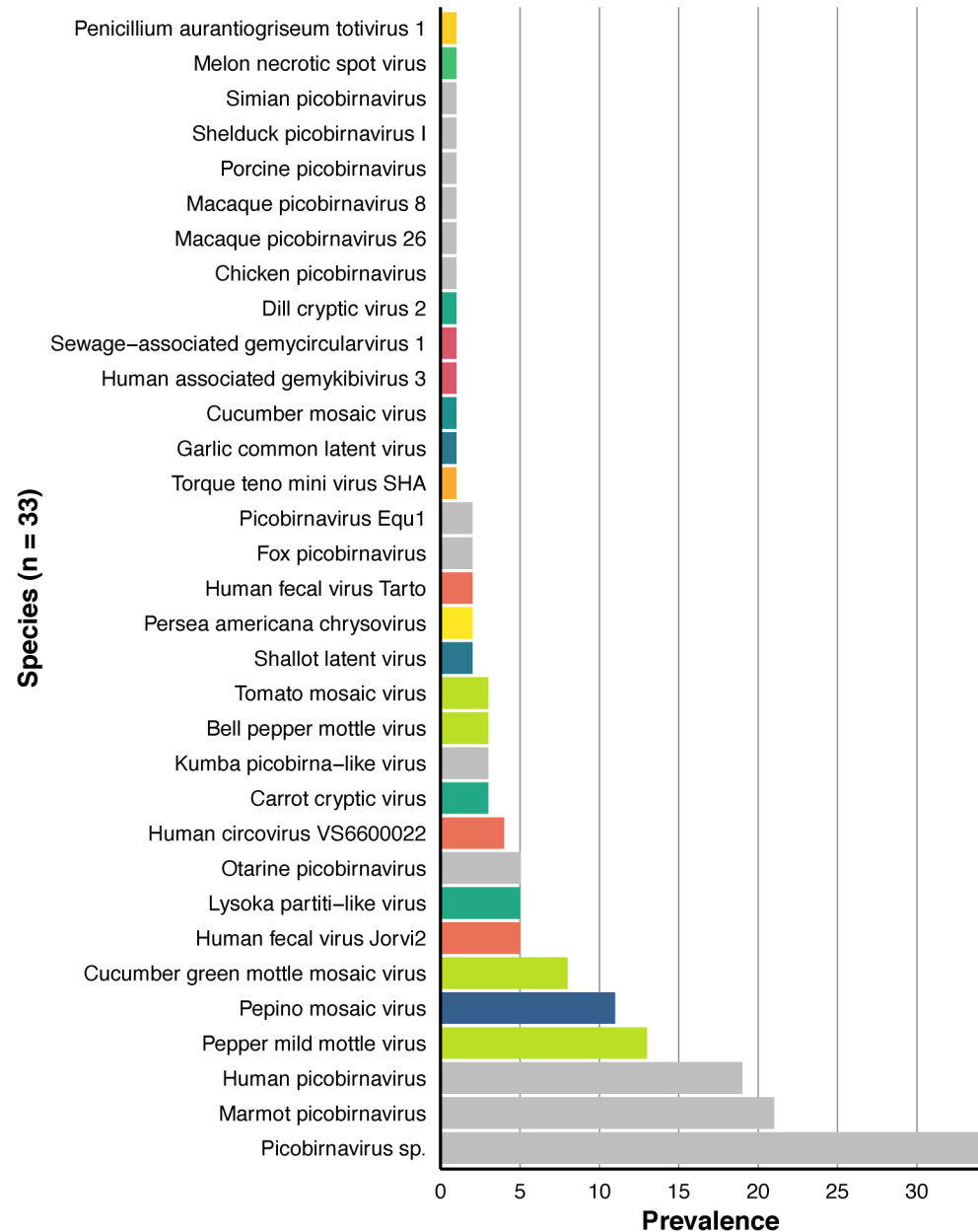

Supplement: FIG S2 [file msystems.00382-21-sf002.pdf]

A

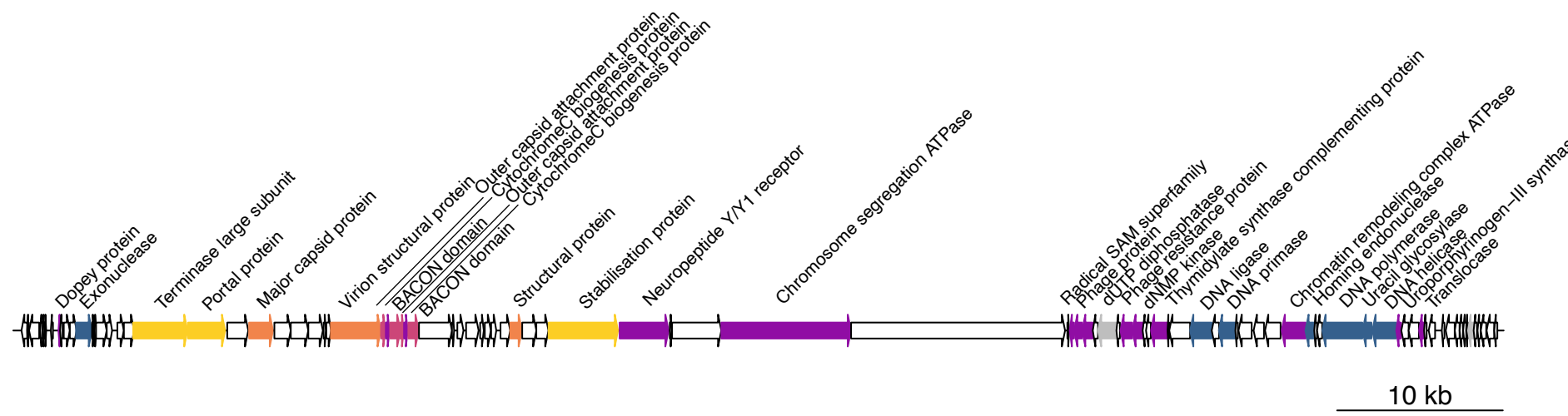

B

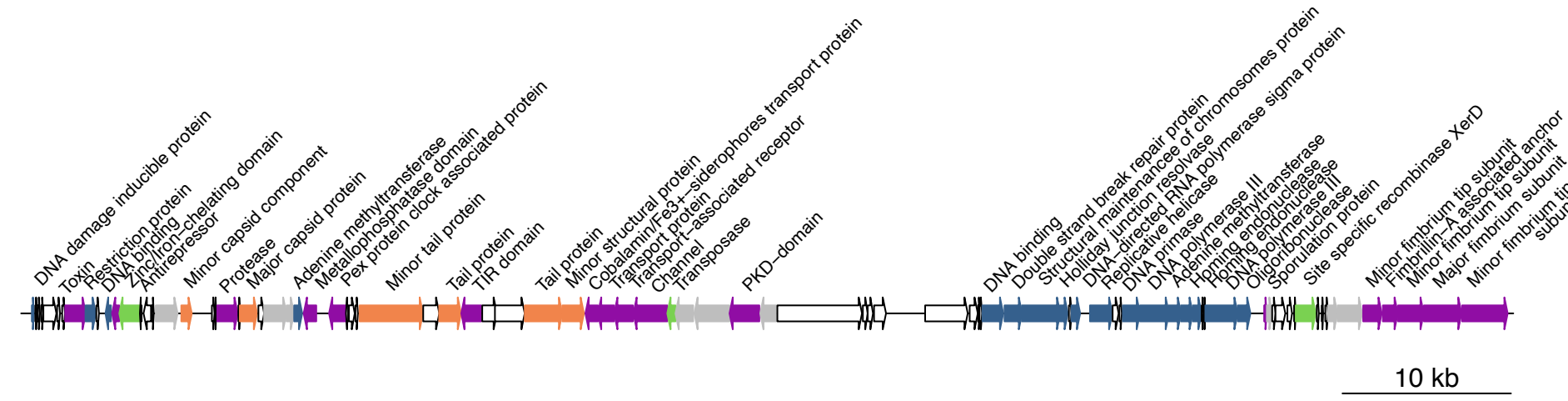

C

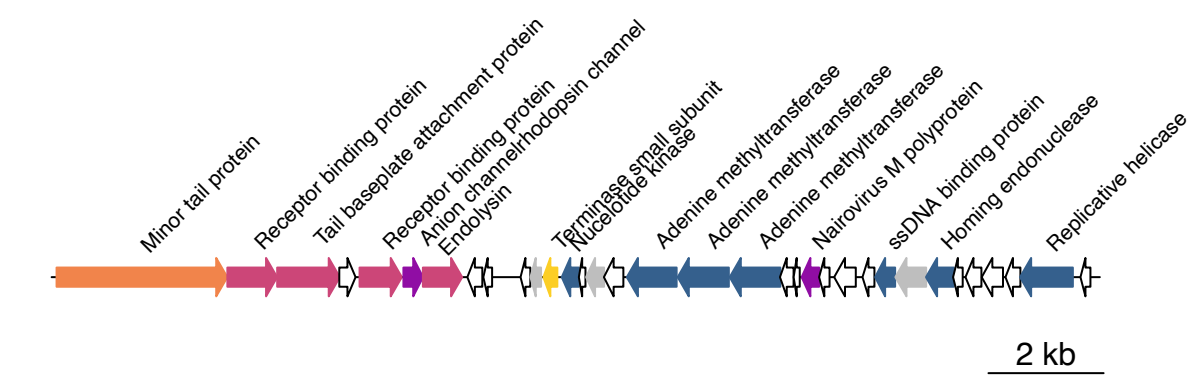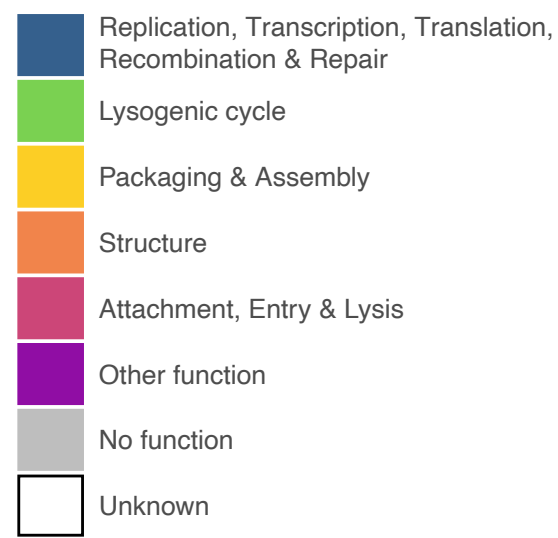

Supplement: FIG S4 [file msystems.00382-21-sf004.pdf]
